# Impact of COVID-19 on the mental health of primary schoolchildren during the later phase of the pandemic: A case report of an 18-month longitudinal survey in a Japanese primary school

**DOI:** 10.1016/j.puhip.2024.100471

**Published:** 2024-01-28

**Authors:** C. Abe, K. Shimatani, K. Tsumura, K. Takaguchi, Y. Nakayama, T. Hayashi, C. Mori, N. Suzuki

**Affiliations:** a Department of Architecture, Division of Creative Engineering, Graduate School of Science and Engineering, Chiba University, 1-33 Yayoi-cho, Inage-ku, Chiba-shi, Chiba, 263-8522, Japan; b Center for Preventive Medical Sciences, Chiba University, 1-33 Yayoi-cho, Inage-ku, Chiba-shi, Chiba, 263-8522, Japan; c Department of Architecture and Urban Science, Graduate School of Engineering, Chiba University, 1-33 Yayoi-cho, Inage-ku, Chiba-shi, Chiba, 263-8522, Japan; d Department of Bioenvironmental Medicine, Graduate School of Medicine, Chiba University, Chiba, Japan, 1-8-1 Inohana, Chuo-ku, Chiba-shi, Chiba, 260-8670, Japan

**Keywords:** Child, COVID-19, Longitudinal studies, Mental health

## Abstract

**Background:**

Drastic changes such as school closures and stay-at-home measures due to the global COVID-19 pandemic, may have long-term negative effects on children's mental health; however, longitudinal studies after 2021 are limited. This study aimed to observe the long-term effects of the COVID-19 pandemic on children's mental health by exploring changes in their mental health over a period of 18 months.

**Study design:**

We conducted a longitudinal study at Chiba Prefecture in Japan, focusing on schoolchildren's mental health changes.

**Methods:**

Data were obtained from the Strengths and Difficulties Questionnaire (SDQ) questionnaire conducted at single primary school three times from October 2021 to March 2023 which and included 183 participants. This study adopted a linear-mixed model to evaluate changes in children's SDQ scores, with sex and grade as the independent variables, and participants as a random effect.

**Results:**

Regarding changes in SDQ scores, there were no significant changes in the total difficulty scores or in each subscale; Emotional Symptoms, Conduct Problems, Hyperactivity/Inattention, Peer Problems, and Prosocial Behavior. There was no statistically significant interaction between changes in SDQ scores and sex.

**Conclusions:**

This report indicates that the impact of the COVID-19 pandemic on the mental health of Japanese primary schoolchildren was negligible in the later phase of the pandemic. However, the impact may differ from country to country owing to factors such as social restrictions during the COVID-19 pandemic.

## 1 Introduction

The COVID-19 pandemic has resulted in significant changes in the social environment. In this context, there has been growing concern that changes such as school closures and staying home may have a negative impact on children's mental health and social development [1]. Several studies have reported that the COVID-19 pandemic has affected children's mental health and social development [2]. In Japan, a study of primary and junior school students reported that children were more hyperactive, inattentive, and less prosocial in May 2020 than in March 2020 [3]. However, these studies were limited to comparisons between the pre-and early pandemic phases in 2020.

Several studies have described the long-term effects of natural disasters on children's mental health. Studies on the Great East Japan Earthquake have reported that some children still suffer from post-traumatic stress disorder and behavioral problems two years after the disaster [4]. Itagaki et al. showed that nuclear accidents and tsunami damage caused by earthquakes may have long-term psychological effects on children [5]. Similar to these natural disasters, the COVID-19 pandemic may also have long-term effects on children's mental health, and children's developmental difficulties may become apparent after some time.

Therefore, we conducted a longitudinal study to observe the long-term effects of the COVID-19 pandemic on children's mental health by exploring changes in children's mental health during the pandemic phase that had been over for more than a year at a time when reports were still limited in Japan.

## 2 Methods

### 2.1 Data collection and participants

For this survey, the three schools were selected based on their geographic proximity to the authors' institutions and their ability to foster cooperative survey efforts, thereby increasing the overall effectiveness of the survey administration. Of the three primary schools in Chiba Prefecture we asked to participate in this study, one school responded. We asked the participating school to fill out a self-administered questionnaire and conducted a longitudinal survey.

The first survey (T1) was conducted in October 2021, the second (T2) in March 2022, and the third (T3) in March 2023. After obtaining written consent from the participants’ guardians, we distributed the questionnaire to two classes of students in grades 1, 3, and 5.

### 2.2 Assessments of mental health

We used the Strengths and Difficulties Questionnaire (SDQ) to assess the mental health of the study participants [6]. The SDQ is a simple questionnaire for screening of children's mental health problems and consists of 25 items in five dimensions, each containing five items: Emotional Symptoms (ES), Conduct Problems (CP), Hyperactivity/Inattention (HI), Peer Problems (PP), and Prosocial Behavior (PB).

Students’ guardians responded to the questions on the SDQ on a three-point scale of 0 (not applicable), 1 (somewhat applicable), and 2 (applicable) regarding whether each item was applicable to their child. Therefore, the total score for each subscale was 0–10 points each, and the total score for the four subscales of difficulty (ES, CP, HI, and PP) was calculated as the total difficulty score (TDS, 0–40 points). Higher scores for these items indicate greater difficulty and are rated negatively. In contrast, higher subscale scores for PB indicate better intensity and are rated positively.

### 2.3 Statistical analysis

We calculated the mean and standard deviation for each time point for the TDS and four subscales: ES, CP, HI, PP, and PB, using the SDQ scores. This study adopted a linear mixed model to evaluate changes in the children's SDQ scores. We used the TDS as the dependent variable; time point as the level 1 independent variable; sex, grade, and the cross-level interaction effect of sex and grade on time point as the level 2 independent variables; and participants as a random effect. The same analysis was conducted with the CP, ES, HI, PP, and PB subscales as the dependent variable. For the independent variables, sex was treated as a categorical variable with 0 for boys and 1 for girls, and grade was treated as an interval variable at each year's level.

Statistical significance was set at p < 0.05. Statistical analyses were performed using the R software (version 4.3.0, https://www.R-project.org/) and the lme4 package. To calculate the P-values, the lmerTest package was used with the Welch-Satterthwait approximate value.

## 3 Results

There were 209 students in the selected classes (6–11 years of age); 183 students (87.6 %) participated in the survey. There were 175 participants (95.6 %) in T1, 165 (90.2 %) in T2, and 158 (86.3 %) in T3. A total of 138 students participated in all three surveys from T1 to T3. The participants consisted of 89 boys and 94 girls, with 64 in first grade, 62 in third grade, and 57 in fifth grade. There were no missing values for sex or grade data. There were no statistically significant differences in sex (p = 0.214) and grade (p = 0.054) between students who fully participated in the survey and those who did not participate or dropped out.

Table 1 shows the SDQ scores (TDS and subscales) at three time points (T1, T2, and T3) and the results of the linear mixed model estimation of the time points (estimate, standard error (SE), and p-value). TDS for T1, T2, and T3 was 7.75, 7.87, and 7.51, respectively, with no significant change from T1 to T3 (estimate ± SE = 0.005 ± 0.354, *p* = 0.988). The CP, ES, HI, PP, and PB subscale scores showed no significant changes.

**Table 1:** SDQ scores in each subcategory from T1 to T3 and estimation results from linear mixed models (*n* = 183).

			T1		T2		T3				
SDQ scores			Mean	(SD)	Mean	(SD)	Mean	(SD)	Estimate	SE	*p* value
	Total difficulty score (TDS)		7.75	(4.8)	7.87	(4.9)	7.51	(4.8)	0.005	0.354	0.988
		Emotional symptoms (ES)	1.59	(1.9)	1.65	(1.9)	1.47	(1.8)	−0.247	0.157	0.117
		Conduct problems (CP)	1.56	(1.5)	1.63	(1.5)	1.55	(1.5)	0.166	0.103	0.110
		Hyperactivity/Inattention (HI)	2.91	(2.2)	2.92	(2.1)	2.68	(2.0)	0.177	0.155	0.257
		Peer problems (PP)^a^	1.67	(1.5)	1.64	(1.3)	1.76	(1.6)	−0.034	0.125	0.786
	Prosocial behavior (PB)		6.68	(2.3)	6.55	(2.3)	6.51	(2.3)	−0.016	0.173	0.927

Abbreviations: SDQ, Strengths and Difficulties Questionnaire; SD, standard deviation; SE, standard error.

a Parameters were estimated using the restricted maximum likelihood estimation method due to nonconvergence only in this model. In the other models, the maximum likelihood method was used.

For the interaction of sex and SDQ score, the slopes of the estimates of fixed effects for TDS were different but not statistically significant (boys = −0.142, girls = 0.194, p = 0.859). For the other subscales, the slopes of the estimates of fixed effects were similar, and no statistically significant sex interaction was observed.

## 4 Discussion

We conducted a longitudinal study of primary school students' mental health for approximately 18 months, from October 2021 to March 2023. There were no significant changes in SDQ scores or negative effects on mental health in either sex. This result is similar to those of previous reports from September 2020 to February 2021 in Japan [7]. This longitudinal study of primary school students was conducted during the early phase of the pandemic, at the time of the declaration of the state of emergency and the implementation of school closures showed no negative effects on children's social behavior, as measured by the SDQ. This previous study was conducted after the Japanese schoolchildren study (May 2020) [3], which found effects on children's emotional/behavioral problems. These findings suggest that the impact of the COVID-19 pandemic on the mental health of Japanese primary school children was negligible in the later phase of the pandemic.

In the UK, longitudinal studies using the SDQ have been conducted from the early 2020 national lockdown period to the relaxation period in 2021. They reported a significant increase in children's mental problems at the initiation of the national lockdown, no such change with during the periods of relaxation, and worsened mental problems during subsequent lockdowns [8]. In response to the COVID-19 pandemic in Japan, the Japanese government declared a state of emergency (semi-lockdown) four times from April 2020 to September 2021 in many districts. At that time, voluntary stay-at-home was required, but it was not forcibly imposed. While long-term school closures restricted children's daily activities, social closures such as stricter lockdowns may contribute more to children's mental health. Our results provide further evidence of the impact of the COVID-19 pandemic on children's mental health, which may vary depending on the country's specific context.

SDQ scores for primary school students as of 2009, considered the Japanese standard, is 7.80 for TDS and 6.22 for PB [9]. Compared to this population, the participants in this survey had similar TDS and slightly higher PB. Therefore, it may be difficult to generalize these findings to primary school students across Japan. In addition, because data on pre-pandemic SDQ scores were not available for study participants, the effects of the pandemic as of October 2021 are unknown and should be interpreted with caution to determine whether it accurately reflects the long-term effects of the pandemic. Due to the lack of detailed information available regarding lifestyle habits and COVID-19 incidence, which might affect the mental health of the participants, we could not include them in our analysis as potential confounders.

In Japan, COVID-19 has been treated as a seasonal influenza virus since May 2023. With further deregulation of social, economic, and other activities, Japan entered a new post-pandemic phase. A recent report on the impact of the COVID-19 pandemic showed that children who had experienced the pandemic (5-year-olds) showed a delay of several months in their development, including communication skills, compared to those who had not [10]. In this survey, we were only able to obtain consent to participate from one school, but further longitudinal studies with longer follow-up periods and more schools are needed to determine the impact of the pandemic on children's mental health and development.

This report shows that the impact on the mental health of Japanese primary school students may be negligible in the late phase of the global COVID-19 pandemic, approximately three years after the event. However, the impact may differ from country to country owing to factors such as social systems during the COVID-19 pandemic.

## Ethical approval

This study was reviewed and approved by the Ethics Committee of Chiba university (approval number: M10085).

## Funding

This research was supported by a Grant-in-Aid for Scientific Research (B) (21H01487). This study was supported by the 10.13039/501100009035JST OPERA Program of Japan (JPMJOP1831). The sponsor has no control over the interpretation, writing, or publication of the manuscript.

## Declaration of competing interest

All authors declare that there is no conflict of interest.
